# Thermal imaging as a tool for studying circadian rhythms in roots

**DOI:** 10.1111/tpj.70992

**Published:** 2026-07-08

**Authors:** Felista Nalufunjo, Omri M. Finkel, Rachel M. Green

**Affiliations:** ^1^ Department of Plant and Environmental Sciences Givat Ram Campus, Hebrew University Jerusalem 9190401 Israel

**Keywords:** Arabidopsis, Barley, Circadian, Microbe, Root, Sorghum, Technical advance, Thermal

## Abstract

Plants rely on the circadian clock to anticipate daily environmental fluctuations and to coordinate key physiological, metabolic, and developmental processes. Most if not all plant cells have semi‐autonomous circadian oscillators. Roots possess a modified yet robust circadian oscillator that is entrained by external cues such as light and temperature to synchronize nutrient uptake, water transport, and metabolic activity. It has been shown that the root and shoot oscillators can communicate through long‐distance signals including mobile proteins and carbon assimilates such as sucrose. Moreover, recent studies indicate root–microbe interactions; root‐associated microbial communities exhibit diurnal oscillations structured by the host circadian system, while microbes can in turn modulate the circadian period and rhythmic outputs of the plant. However, in general, while the shoot circadian oscillator has been extensively characterized, much less is known about the root circadian system. Progress has been hampered by a lack of high‐throughput, non‐invasive methods to study root rhythmicity. Existing methods including luciferase reporters, quantitative RT‐PCR, and microscopy remain limited by cost, destructive sampling, or require transgenic lines with reporter genes. We have developed a thermal infrared imaging platform that enables non‐invasive, high resolution of circadian rhythms in roots across plant species and growth conditions. We show that our system can be used to analyse metabolite and microbial effects on root circadian regulation. This platform provides new opportunities to investigate below‐ground circadian regulation and the possibilities of harnessing the root clock to enhance plant performance and resilience.

## Significance Statement

Circadian studies in plants have largely focused on shoots, leaving the root clock and its role in plant–environment interactions poorly understood. We present a thermal infrared imaging platform that enables high resolution, non‐destructive analysis of circadian rhythms in plant roots without transgenic reporters, facilitating investigation of below‐ground circadian regulation and its modulation by metabolites and microbial interactions.

## INTRODUCTION

Like almost all organisms, plants experience daily environmental oscillations and have evolved circadian systems to anticipate and adapt to these changes. Plant circadian systems consist of oscillators, input pathways that synchronize the oscillator with environmental and cellular states, and output pathways that regulate diverse metabolic, physiological, and developmental processes. These include gene expression, chlorophyll biosynthesis, starch metabolism, stress responses, and growth (Creux & Harmer, 2019). Given the diversity of processes regulated by the circadian system in plants, it is not surprising that a robust, synchronized circadian system may enhance plant growth and vitality (Oravec & Greenham, 2022).

Over recent decades, the oscillator mechanism that generates circadian rhythms has been extensively studied in *Arabidopsis thaliana* (Arabidopsis) and shown to be based on a network of feedback loops of components, mostly transcription factors, which regulate each other's expression (Nakamichi, 2020). At the core of the Arabidopsis oscillator mechanism, expression of *CIRCADIAN CLOCK‐ASSOCIATED 1* (*CCA1*) and *LATE ELONGATED HYPOCOTYL* (*LHY*) peaks around dawn. CCA1 and LHY regulate the expression of daytime‐phased genes *PSEUDO‐ RESPONSE REGULATORs* (*PRRs*) and evening‐phased genes, *TIMING OF CAB EXPRESSION 1* (*TOC1*), *GIGANTEA* (*GI*), and elements of the evening complex (EC); *LUX ARRHYTHMO* (*LUX*), *EARLY FLOWERING 3* (*ELF3*), and *ELF4*. The expression of *CCA1* and *LHY* is in turn regulated by TOC1, CHE (CCA1 HIKING EXPEDITION), and PRRs. Post‐translational regulation also plays a crucial role in the oscillator; for example, ZEITLUPE (ZTL), an F‐box protein and photoreceptor with E3 ubiquitin ligase targets, TOC1 and PRR5 for degradation (Pérez‐Llorca & Müller, 2024).

Although roots, often referred to as the ‘hidden half’ of the plant, play vital roles in anchoring, water and nutrient absorption, food storage, and enhancing plant performance under both optimal and stressful conditions (Gowda et al., 2011; Kalra et al., 2024; Vives‐Peris et al., 2020), they are more challenging to study than the aerial parts of the plant and are less well understood. Similarly, relatively little is known about the root circadian clock, although studies have shown that circadian rhythms in roots are robust and that the clock is involved in regulating diverse root functions (Beeckman & Eshel, 2024). The root oscillator appears to be a modified version of the shoot oscillator and is also entrained by external cues like temperature and light via phytochrome B (Bordage et al., 2016; James et al., 2008; Nimmo et al., 2020). Moreover, shoot and root circadian rhythms exhibit mutual influence; for instance, shoot oscillators affect root rhythms via ELF4 signaling and sucrose transport, while root oscillators contribute to shoot rhythm precision by regulating potassium transport (Chen et al., 2020; Uemoto et al., 2023). Root circadian systems also exhibit significant autonomy (Greenwood et al., 2022; Thain et al., 2000). One study has indicated that Arabidopsis root tips have shorter circadian periods than the rest of the root (Gould et al., 2018), however, roots generally exhibit longer free‐running periods than shoots (Greenwood et al., 2019).

Numerous studies have highlighted the mutual influences between roots and microorganisms in the rhizosphere (Balestrini et al., 2024; Demiwal et al., 2024; Durán et al., 2018; Newman et al., 2022). These interactions enhance plant growth by improving soil chemistry, structure, and nutrient availability, while also bolstering resistance to diseases and environmental stresses. Rhizosphere bacterial communities are subject to diurnal control (Staley et al., 2017) and are influenced by the plant's circadian system; for example, mutations in *LHY* reduce root nodule formation in *Medicago truncatula* (Kong et al., 2020), and *Arabidopsis* plants with *toc1* and *ztl* mutations exhibit significantly altered rhizosphere communities compared with wild‐types (Hubbard et al., 2018). Interestingly, rhizosphere microbes may also impact the circadian period of their host plant (Hubbard et al., 2021). However, the effects of specific microbial taxa on plant circadian rhythms remain largely unexplored.

Although the root circadian system plays a key role in coordinating below‐ground processes and holds potential for improving crop resilience and optimizing resource use, research on the root circadian clock has progressed relatively slowly. This is largely due to a lack of high‐throughput, non‐invasive analysis methods for analyzing root rhythmicity. Existing techniques such as luciferase imaging, gene expression analysis using quantitative reverse‐transcription PCR (qRT‐PCR) and microscopy (Nimmo et al., 2020; Perez‐Garcia et al., 2022; Perianez‐Rodriguez et al., 2021) have notable limitations. For example, luciferase imaging requires transgenic plants and costly reagents, and qRT‐PCR and transcriptome analyses are labor intensive and destructive. Thermal (infrared, IR) imaging has previously been used to study root architecture and growth (Bizet et al., 2017; Shi et al., 2018) and we have shown that thermal imaging can be applied to study circadian traits in the aerial parts of plants (Dakhiya & Green, 2019). In this article, we show how we can adapt thermal imaging to give us a high resolution, non‐invasive platform for studying root rhythms. We show that the technique is suitable for roots from different plant species grown under diverse conditions and can be used to explore the effects of metabolites and microbes on the root circadian system. While with the system we report in this article we can measure 50 Arabidopsis plants at a time, we predict that it would not be difficult to scale up to much higher throughput experiments.

## RESULTS

### Arabidopsis root temperature is under circadian and diel control

**Figure 1:**
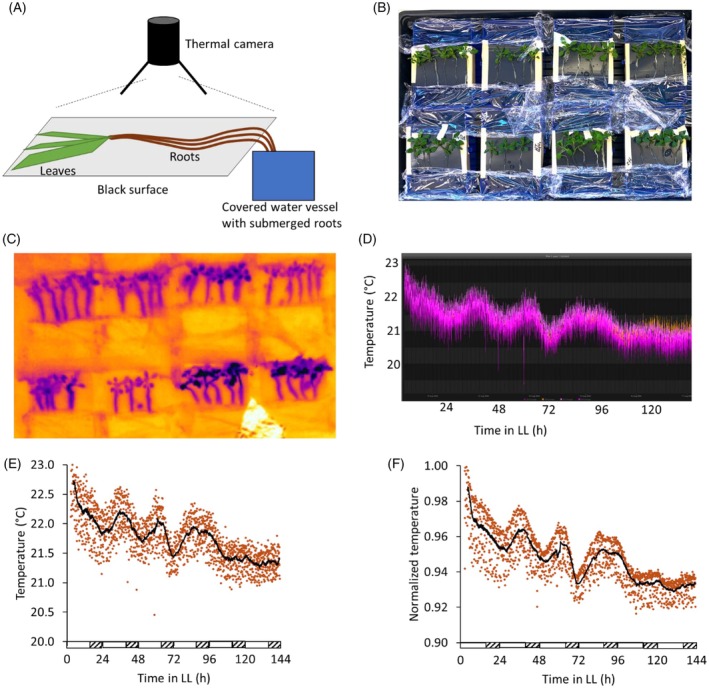
Thermal imaging of root rhythmicity. (A) Root thermal imaging platform. The thermal camera is positioned in the growth chamber above the plants to capture temperature changes on surfaces of leaves and roots. (B) White light image and (C) thermal image of 3‐week‐old Arabidopsis plants on a black background. The root tips are dipped in water which is covered by plastic wrap and secured with adhesive tape. (D–F) Thermal oscillations in roots of Arabidopsis plants grown for 3 weeks in LD and then transferred to LL. (D) Individual rhythmic traces of root temperature measurements from four individual WT plants monitored over 6 days. (E) Average and (F) Normalized average root temperature relative to the aluminum foil control. The smoothed black traces represent 24‐point moving averages of eight measurement points while the brown dots show the temperature of each measurement at each timepoint.

Our first goal was to determine whether root temperatures showed oscillations. Wild type (WT) Arabidopsis plants were grown on MS/agar media in 14:10 Light‐Dark (LD) conditions for 3 weeks before being removed from the medium. The roots were washed and placed on a black surface with the root tips submerged in water (Figure 1A,B) as described in the Materials and Methods. Thermal images were captured every 5 minutes in LD conditions using a FLIR‐05A305sc thermal imaging camera and showed clear temperature oscillations (Figure 1C–E). To control for temperature fluctuations in the chamber, we normalized the thermal readings to an aluminium foil reference placed 10 cm from the plants (Figure 1E).

**Figure 2:**
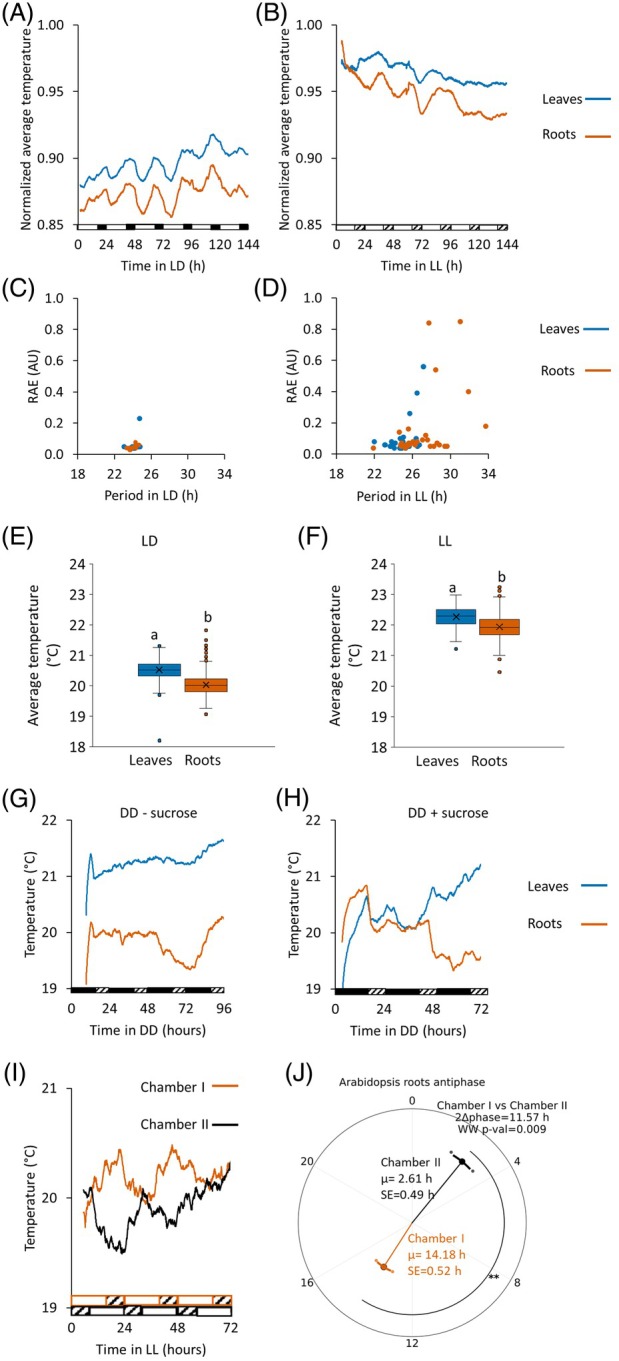
Root temperature is under diel and circadian control in Arabidopsis. WT Arabidopsis plants were grown on ½ MS medium without sucrose for 3 weeks under LD at 22°C before imaging under LD, LL, or DD conditions. For each plant, 1–3 leaf and root regions were measured. (A, B) Normalized thermal rhythms of eight root (orange) and leaf (blue) samples under LD and LL. Data in (B, D, and F) are from the results shown in Figure 1(E,F). LD data were pooled from three independent experiments (*n* = 3), comprising 7–11 samples per replicate (total: 29 roots and 23 leaves). LL data were pooled from five independent experiments (*n* = 5), each containing 6–8 samples (total: 34 roots, 30 leaves). Individual root and leaf rhythms were extracted, with statistical comparisons based on experimental‐level means. (C, D) Relative amplitude error (RAE) versus period length for roots and leaves under LD (C) and LL (D). Data points represent individual samples. (E, F) Mean leaf and root temperatures across the first three measurement days under LD (E) or LL (F); *n* = 2 independent experiments. Different letters indicate significant differences (Student's *t*‐test, *P* < 0.001). (G, H) Thermal rhythms in DD without (G) or with (H) 0.5% sucrose; *n* = 2 (G) and *n* = 3 (H) independent experiments, 2–4 samples per experiment. White, black, and hatched bars indicate light, dark, and subjective night. (I, J) Circadian phase differences between Arabidopsis roots grown in antiphase LD conditions in Chamber I (07:00–21:00) and Chamber II (19:00–09:00) were analyzed after transfer to constant light (LL), when roots were imaged simultaneously. (I) Thermal circadian cycles of seven individual roots from Chamber I and six roots from Chamber II. (J) Mean phase angles of roots from the two chambers were compared using the Watson–Williams (WW) test (*P* < 0.01), *n* = 2 independent repeats. The absolute circular difference between mean phases (2Δphase, in hours) determined the magnitude of phase separation. The ‘WW *P*‐value’ indicates the statistical difference of mean phase angles between roots from chamber I and chamber II obtained from the WW test. ‘μ’ denotes the mean phase of roots in each chamber, and ‘SE’ denotes the standard error of each mean.

Having shown that root temperature oscillates (Figure 1D–F), our next goal was to determine whether thermal imaging could be used to accurately assay circadian and diel rhythm traits. WT Arabidopsis plants were grown in LD conditions for 3 weeks then transferred to the customized growth chamber with the thermal camera in LD, LL, or DD and the average diel and circadian temperatures of the leaves and roots imaged. Figure 2(A) shows that the normalized diel periods of Arabidopsis roots and leaves were 24.1 and 24.2 h. By contrast, in LL, consistent with previously published results (Bordage et al., 2016; James et al., 2008), the roots displayed a significantly longer period than the leaves by ~2.4 h (*P* < 0.05) (Figure 2B). Although they showed more variation in LL (Figure 2C,D), both organs maintained robust rhythms with average RAE of 0.2 for the roots and 0.1 for the leaves. In LD, roots and leaves were 100% rhythmic while in LL roots were 85% rhythmic and leaves were 88% rhythmic. In both LD and LL conditions, the average temperatures over the first 3 days of measurement indicated that overall, roots were cooler than leaves (*P* < 0.001), but both roots and leaves exhibited higher temperatures in LL than LD (*P* < 0.001) (Figure 2E,F).

Consistent with reports that rhythmicity is affected under constant dark (DD) conditions (Dalchau et al., 2011), we observed that thermal rhythms in both roots and leaves dampened rapidly in DD (Figure 2G) and rhythmicity was observed in only 6.7% of roots and 18% of leaves. Exogenous sucrose can support circadian rhythms in constant darkness (DD) (Dalchau et al., 2011; Li et al., 2022). We therefore tested the effect of supplementing the root‐immersion water with 0.5% sucrose. In DD + sucrose, 100% of roots and 78.6% of leaves exhibited rhythms that dampened after 48 h, confirming the role of sucrose as a metabolic signal that supports circadian rhythms (Figure 2H). Notably, in DD + sucrose, root rhythms had significantly a lower RAE (RAE = 0.07) than in DD without sucrose (RAE = 0.17); (*P* < 0.01). Interestingly in DD + sucrose we did not observe statistically significant period differences between roots (25.9 h) and leaves (27.3 h).

Finally, to confirm that the root rhythms we detected are not caused by changes in the local environmental conditions, we entrained two batches of Arabidopsis in opposite LD cycles; in Chamber I, lights were turned on at 7:00 (ZT0) and turned off at 21:00, while in Chamber II, lights were turned on at 19:00 (ZT0) and turned off at 9:00 the next day. After 3 weeks in LD, the plants were transferred to LL and imaged. Figure 2(I,J) show that the plants entrained in these antiphase photoperiods continue to show antiphasic circadian oscillations in LL with a phase difference of ~11.57 h (*P* < 0.01; Watson–William's test). Together with the very low fluctuations in the temperature of the aluminum foil control in the chamber (Figure S1), our results confirm that root thermal rhythms we observed are caused by changes in root temperatures and not driven by changes in environmental temperatures.

### Root thermal rhythms in Arabidopsis circadian mutants

**Figure 3:**
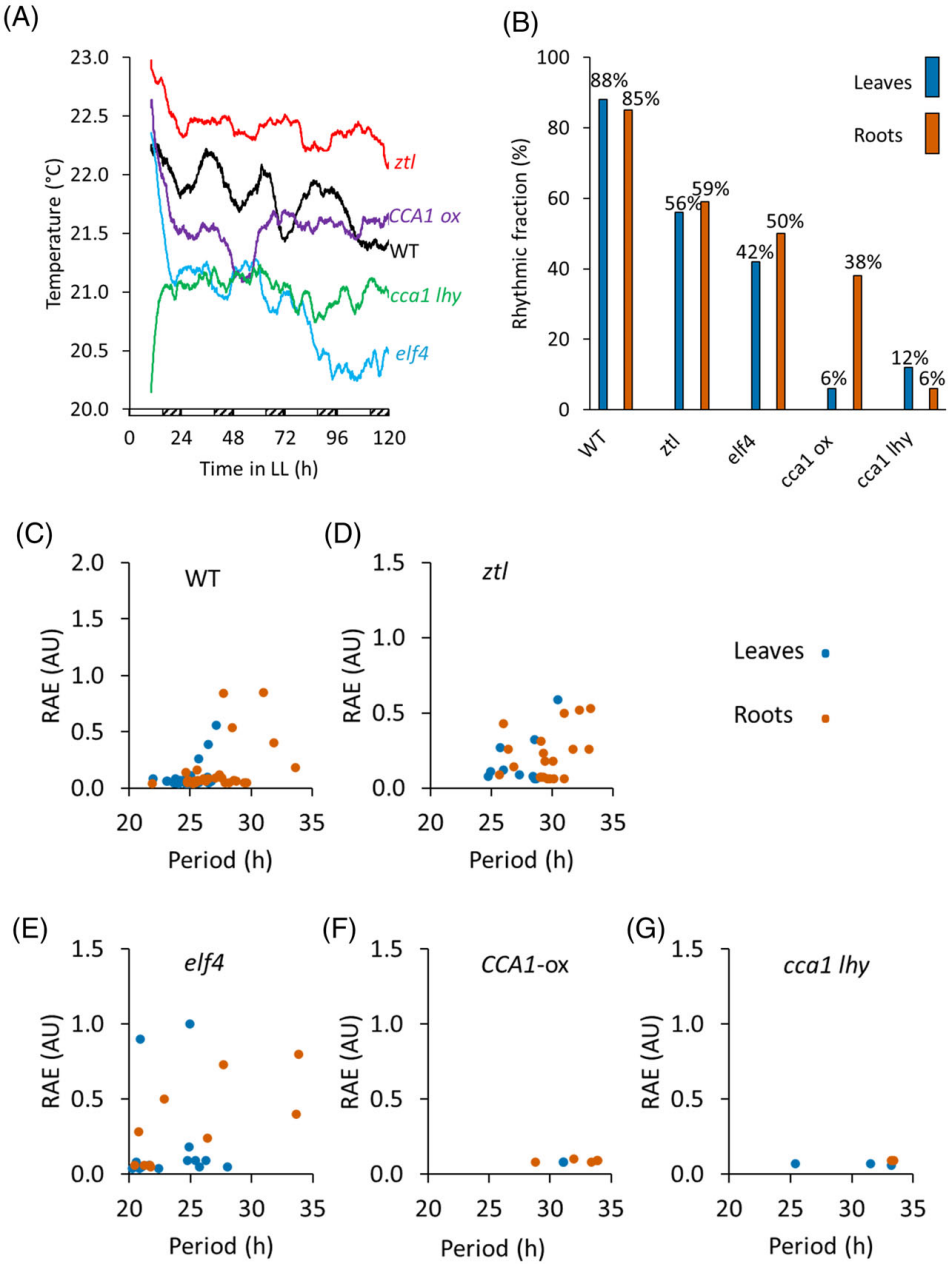
Circadian mutants affect thermal circadian rhythms in Arabidopsis roots. WT, *ztl*, *elf4*, *cca1 lhy*, and *CCA1*‐ox Arabidopsis plants were grown under 14:10 h light:dark cycles and transferred to constant light (LL) for thermal imaging of leaf and root rhythms. Measurements were taken from 1 to 3 randomly selected regions of leaves or roots per plant. (A) Rhythmic root traces represent the mean of 8 (WT), 6 (*ztl*), 5 (*elf4*), 7 (*CCA1*‐ox), and 9 (*cca1 lhy*) samples pooled across two independent experiments and smoothed with a 24‐point moving average. WT root data are from the results shown in Figure 1(E,F). (B) Rhythmic fractions of leaves (blue) and roots (orange) by genotype. (C–G) Relative amplitude error (RAE) plotted against circadian period for (C) WT, (D) *ztl*, (E) *elf4*, (F) *CCA1*‐ox, and (G) *cca1 lhy*. Circadian period values were extracted for individual roots and leaves across 3–5 independent experiments per genotype. Data points in the dot plots represent individual samples. Statistical analyses were performed at the experiment level.

Having shown that our root thermal imaging technique can be used to analyze diel and circadian rhythms, we tested the technique on roots of several Arabidopsis circadian oscillator mutants, *ztl‐3* (SALK_069091), *elf4* (CS_9386), *CCA1‐*ox and the *cca1 lhy* double mutant (SALK_031092). WT and mutant plants were grown in LD for 3 weeks then imaged in LL. Figure 3(A) shows that root thermal oscillations were affected in the mutants. All mutants exhibited a lower RF compared with WT (Table 1; Figure 3B). Consistent with previous reports (Li et al., 2020) that *ZTL* contributes to both root and leaf circadian oscillations, the thermal circadian periods of the *ztl* mutant roots and leaves were significantly longer (~2.2 and ~2.1 h, respectively) than WT (Figure 3C,D) (*P* < 0.05). In contrast, the *elf4* mutant roots and leaves exhibited a significantly shorter period than WT roots and leaves by ~5 and ~2.7 h respectively (*P* < 0.05) (Figure 3C,E). Notably, the *elf4* leaves and roots no longer displayed the significant period differences observed in WT (*P* = 0.91). The *CCA1‐*ox (Figure 3F) and *cca1 lhy* double mutant (Figure 3G) largely displayed loss of both leaf and root rhythmicity.

**Table 1:** Circadian periods and RAE of leaves and roots of WT and mutant plants in LL

	Leaves		Roots	
	Period (h) ± SEM	RAE (AU) ± SEM	Period (h) ± SEM	RAE (AU) ± S.E.M
WT	24.9 ± 0.4	0.1 ± 0.03	27.3 ± 0.5	0.2 ± 0.07
*ztl‐3*	27.0 ± 0.4	0.2 ± 0.04	29.5 ± 0.4	0.2 ± 0.06
*elf4*	22.2 ± 0.6	0.5 ± 0.5	22.3^a^	0.25^a^
*CCA1*‐ox	31.1^a^	0.08^a^	32.4^a^	0.10^a^
*cca1 lhy*	30.0^a^	0.07^a^	33.3^a^	0.10^a^

^a^ Values from lines that showed rhythmic behavior in only one experimental repeat.

### Thermal imaging reveals root rhythms across diverse plant species

**Figure 4:**
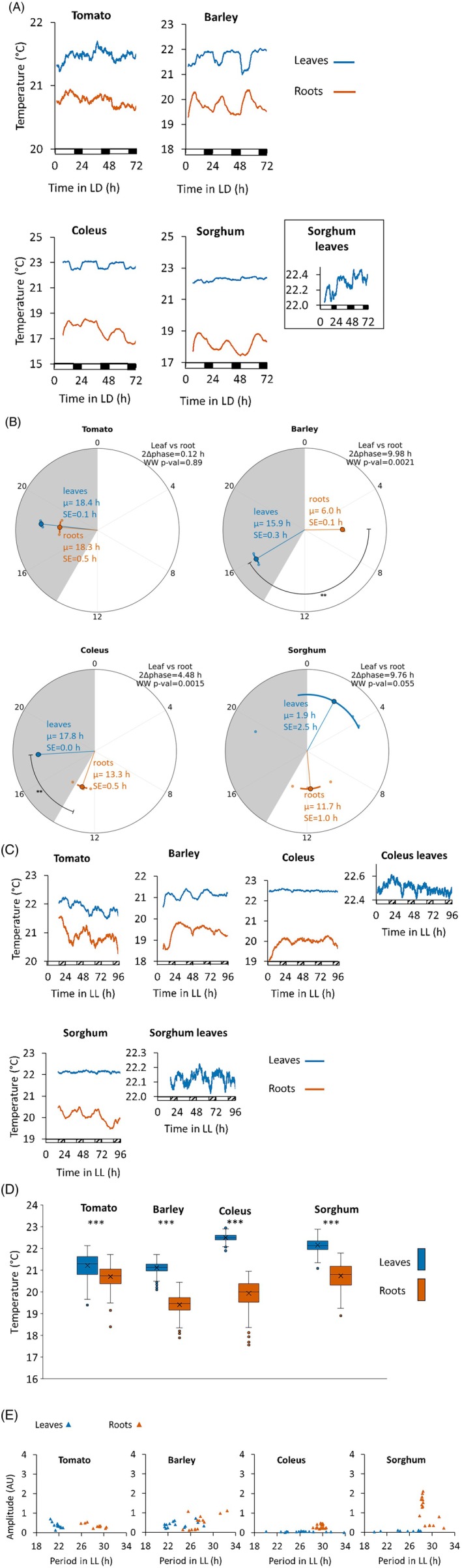
Thermal rhythms revealed in tomato, barley, coleus, and sorghum. Plants were grown under LD conditions and imaged for 3 days in LD (A, B) or 4 days in LL (C–E). For each plant, 1–3 regions of leaves or roots were measured. (A) Rhythmic traces representing the average of eight measurement points per organ (roots and leaves), pooled across two independent experimental runs per species, and smoothed using a 24‐point moving average. (B) Leaf–root circadian phase differences in LD were analyzed using the Watson–Williams (WW) test to compare mean phase angles between leaves and roots. The absolute circular difference between mean phases (2Δphase, in hours) was calculated to quantify the magnitude of phase separation. The ‘WW *P*‐value’ indicates the statistical difference between mean phase angles of tissues of each plant obtained from the Watson–Williams test. ‘μ’ represents the average phase of each tissue, and ‘SE’ denotes the standard error of each mean. (C) Rhythmic traces in LL, shown as the average of eight measurement points per organ, pooled across two independent experimental runs per species, and smoothed using a 24‐point moving average. (D) Mean leaf and root temperatures in LL over 3 days. (E) Amplitude versus period in LL. Each point represents an individual leaf (blue) or root (orange) measurement pooled across independent runs. (D, E) Data derived from 2 to 3 independent experimental runs per species, of 2–12 leaf or root samples per experiment. White, black, and hatched bars indicate light, dark, and subjective night, respectively. (D) Statistical significance was assessed using Student's *t*‐test (***P ≤ 0.001).

Our next goal was to test whether we could use the imaging platform for different plant species. We chose *Solanum lycopersicum* (tomato) as a model dicotyledonous C3 crop plant, *Hordeum vulgare* (barley) and *Sorghum bicolor* (sorghum) as monocotyledonous C3 and C4 crop plants, with fibrous root systems, and *Coleus blumei* (coleus) for its pigmented leaves, which are rich in secondary metabolites such as anthocyanins. The plants were grown as described in “Materials and Methods” section for 4 weeks in LD at 22°C before being imaged either in LD or in LL for 3–5 days. Figure 4(A) shows that roots and leaves of all four species were rhythmic in LD with similar diel periods. However, there were considerable inter‐species variations in rhythmic traits; tomato and sorghum roots and leaves exhibited similar phased oscillations in LD, while for barley and coleus, there were significant phase differences between roots and leaves (*P* < 0.01, Watson–William's test) (Figure 4B). Figure 4(C–E) demonstrates that temperature oscillations were detectable in roots of all four species under LL conditions. As observed in Arabidopsis, the roots in these other species oscillated at a lower mean temperature than their aerial tissues (Figure 4C,D). Across all tested species, roots consistently exhibited a longer circadian period than leaves; however, the specific period lengths and amplitude varied between species (Figure 4E; Table 2). Both roots and leaves were robustly rhythmic in LD and LL with average RAE values well below 0.6 (Table 2).

**Table 2:** Average period, RAE, and amplitude of thermal rhythms in leaves and roots of tomato, barley, coleus, and sorghum in (i) LD and (ii) LL

(i) Parameter in LD	Tomato		Barley		Coleus		Sorghum	
	R	L	R	L	R	L	R	L
Period (h)	24.1	23.0	24.7	24.4	24.6	23.9	25.3	25.7
± SEM	±0.8	±1.5	±0.09	±0.09	±0.7	±0.06	±0.3	±0.4
RAE (AU)	0.08	0.2	0.07	0.06	0.12	0.06	0.08	0.08
± SEM	±0.01	±0.1	±0.0001	±0.001	±0.07	±0.0005	±0.02	±0.02
Amplitude (AU)	0.7	0.6	1.1	1.4	1.0	2.2	1.4	0.3
± SEM	±0.2	±0.5	±0.5	±0.2	±0.05	±0.3	±0.6	±0.05

(ii) Parameter in LL	Tomato		Barley		Coleus		Sorghum	
	R	L	R	L	R	L	R	L
Period (h)	27.9	21.3	27.8	23.7	29.7	26.4	28.5	24.2
± SEM	±1.4	±0.6	±0.9	±0.14	±0.1	±0.1	±0.8	±0.6
RAE (AU)	0.06	0.006	0.08	0.07	0.1	0.3	0.3	0.4
± SEM	±0.002	±0.02	±0.004	±0.01	±0.03	±0.1	±0.1	±0.2
Amplitude (AU)	0.4	0.5	0.6	0.4	0.3	0.05	0.9	0.09
± SEM	±0.1	±0.2	±0.2	±0.01	±0.1	±0.0	±0.6	±0.02

L, leaf samples; R, root samples.

### Detached roots maintain thermal circadian rhythms

**Figure 5:**
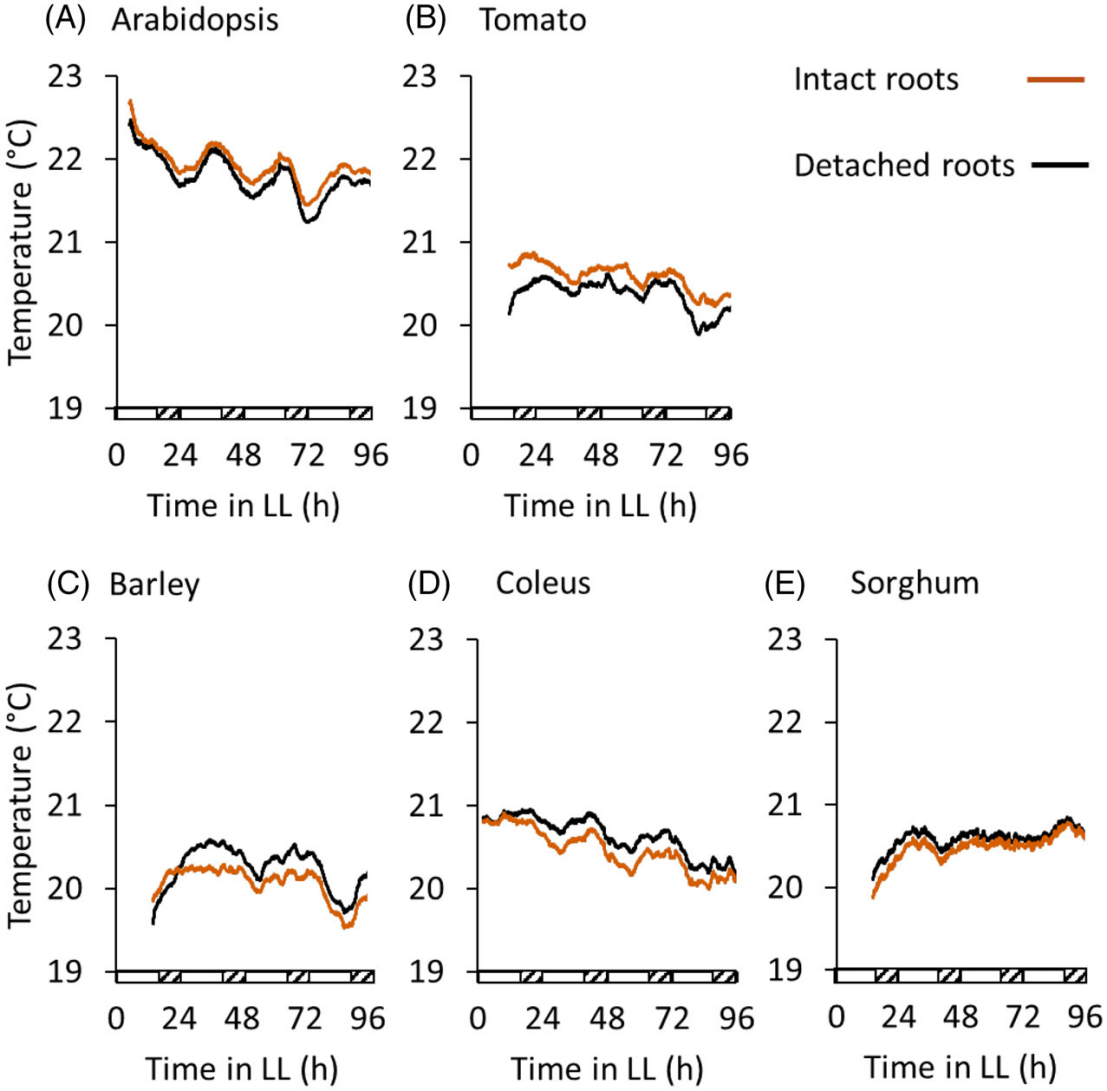
Detached roots retain circadian rhythmicity. Plants were grown under LD conditions at 22°C for 3 weeks (Arabidopsis) or 4 weeks (tomato, barley, coleus, and sorghum). Roots were detached from some plants and, together with the remaining intact plants, transferred to the thermal imaging chamber for imaging in LL. For each plant, 1–3 randomly selected root regions were measured per plant. Panels (A)–(E) show representative thermal rhythmic profiles of five intact (brown) and five detached (black) roots combined from two independent repeats of (A) Arabidopsis, (B) tomato, (D) coleus, and (E) sorghum, and three independent repeats in (C) barley.

Our observations that roots consistently exhibited longer circadian periods than leaves in LL (Table 2ii), and that root and leaf rhythms can be antiphasic (Figure 4) indicate that root thermal oscillations are at least partially autonomous of the aerial parts of the plant. To test this autonomy directly, we compared thermal rhythms in intact and detached roots of Arabidopsis, tomato, coleus, sorghum, and barley. Plants were entrained in LD, and then thermal rhythms of roots from 3‐week‐old Arabidopsis and 4‐week‐old tomato, coleus, sorghum, and barley were recorded in LL at 22°C either while attached to the shoot or after detachment (as described in “Materials and Methods” section). As shown in Figure 5 and Table 3, detached roots maintained circadian periods like those of intact roots. Surprisingly, detached roots of tomato, barley, coleus, and sorghum tended to be even more robustly rhythmic than intact roots with lower RAEs and higher RFs. This enhanced rhythmicity suggests that signals from the shoot such as sucrose and light piping (James et al., 2008; Kircher & Schopfer, 2023; Nimmo et al., 2020) may be modulating endogenous root rhythms. Taken together, our results demonstrate that thermal imaging is a powerful and versatile platform for analyzing circadian rhythms across diverse plant species.

**Table 3:** Average circadian period ± standard error of the mean, RAE, and rhythmic fractions of detached and intact roots in LL

Plant roots	Intact roots			Detached roots		
	Average period (h)	Average RAE (AU)	Rhythmic fraction (%)	Average period (h)	Average RAE (AU)	Rhythmic fraction (%)
Arabidopsis	27.3 ± 0.5	0.20 ± 0.07	88	28.0 ± 0.3	0.06 ± 0.01	73
Tomato	27.9 ± 1.4	0.06 ± 0.002	71	28.3 ± 1.1	0.10 ± 0.05	78
Barley	27.8 ± 0.9	0.06 ± 0.03	85	27.7 ± 0.9	0.0 ± 0.004	99
Coleus	29.7 ± 0.1	0.12 ± 0.07	87	29.5 ± 0.36	0.06 ± 0.0005	100
Sorghum	28.5 ± 0.8	0.08 ± 0.02	61.4	29.6 ± 0.5	0.06 ± 0.007	91

Data are from 2 to 5 independent experimental repeats of intact and detached roots of tomatoes, barley, coleus, and sorghum. The data for intact Arabidopsis roots is identical to that presented in Table 1, while data for the other intact plant species correspond to those in Table 2(ii) with the experiments conducted concurrently.

### Measuring the effects of microbes on root thermal rhythms

**Figure 6:**
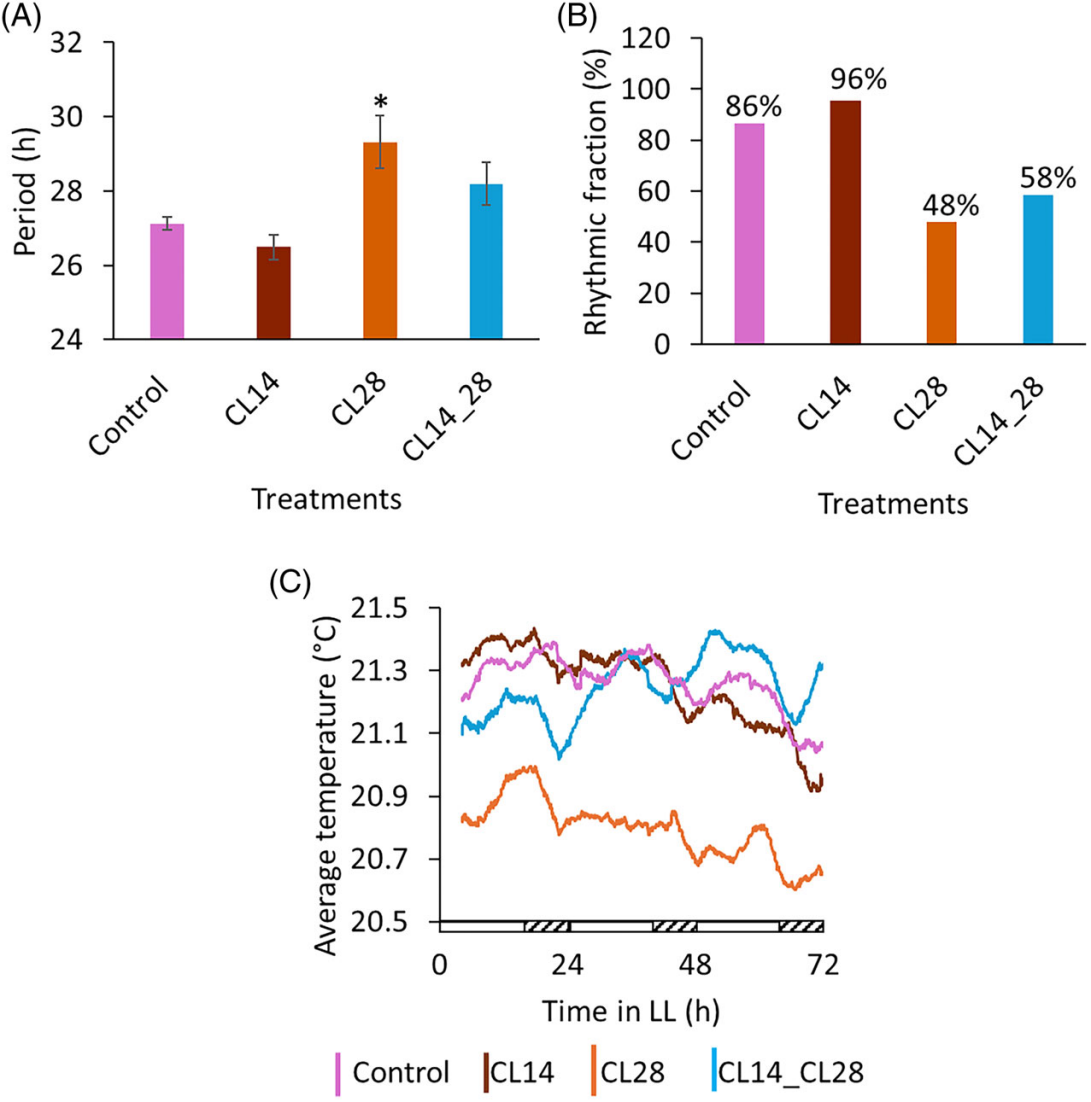
Thermal imaging reveals the impact of CL14 and CL28 bacterial strains on circadian rhythms in Arabidopsis roots. Two‐week‐old WT Arabidopsis were transferred to plates containing *Variovorax* CL14 and *Arthrobacter* CL28 strains individually or in combination, or MgCl_2_ control and incubated in LD for 6 days. Thermal images were acquired over 3 days under constant light (LL). For each plant, 1–2 randomly selected regions of interest from roots were analyzed. (A) Average circadian period, (B) rhythmic fraction, and (C) thermal cycles of roots with or without bacterial treatments. (A) Statistical analysis was performed using one‐way ANOVA followed by Dunnett's post hoc test comparing each treatment to the MgCl_2_ control where (*) is *P* < 0.05. Data were obtained from three independent experimental runs (*n* = 3). All plants included in the analysis were assessed for rhythmicity, and the number of plants exhibiting rhythmic behaviur per treatment per run ranged from 2 to 6. (C) Data from 6 to 13 plants were smoothed using a 24‐point moving average. White and hatched bars indicate light and subjective dark, respectively.

There is evidence that microbial communities can influence plant circadian rhythms (Hubbard et al., 2021; Li et al., 2018) yet the specific contributions of individual microbial taxa to clock function remain almost completely unknown. We tested whether our thermal imaging technique could be used to evaluate the effects of microbes on root circadian rhythms. *Arthrobacter* and *Variovorax* are widespread bacterial strains that interact with and affect root growth. In synthetic bacterial communities, *Variovorax* strain CL14 can reverse root growth inhibition (RGI) caused by *Arthrobacter* strain CL28 through an auxin‐degradation operon (Finkel et al., 2020; Qi et al., 2022). Given the connections between auxin signaling and the circadian clock in *Arabidopsis* (Covington & Harmer, 2007), we reasoned that *Arthrobacter* and *Variovorax* might influence circadian regulation in roots. Two‐week‐old WT Arabidopsis were transferred to plates with bacteria or MgCl_2_ (as described in “Materials and Methods” section) then incubated in LD for 6 days before imaging in LL. Figure 6 shows that CL28 significantly affected root circadian rhythms, not only causing an increase in period (*P* < 0.05), but also reducing the percentage of plants that showed rhythmicity to 48%. Co‐application of CL14 restored the circadian period to WT levels and increased the rhythmic fraction by 10% relative to CL28 alone. Roots treated with CL14 alone exhibited the highest rhythmic fraction at 96%, comparable to 86% in control roots. Thus, individual microbial taxa exert distinct effects on circadian traits, and their combined action can produce synergistic alterations in rhythmicity. Taken together, our results show that thermal imaging is a powerful tool for assessing how microbes shape root circadian rhythms.

### Root thermal rhythms may be driven by changes in root water content

Our final goal was to start to understand the basis for the thermal circadian rhythmicity in roots. Since the circadian periods of root and leaf rhythms differ and detached roots are robustly rhythmic, root temperature oscillations cannot be attributed solely to transpiration from the aerial tissues. We hypothesized that the circadian and diel oscillations in root temperature we observed may be driven by fluctuations in root water content. Roots absorb water mainly by osmosis, driven by water potential gradients, with uptake enhanced by root hairs and aquaporins (Bhatla & Kathpalia, 2023; Cai & Ahmed, 2022; Steudle, 2000). Roots also redistribute water passively from wetter to drier soil layers, especially at night when transpiration is low (Neumann & Cardon, 2012). These dynamics influence root water content and may affect the root temperature.

**Figure 7:**
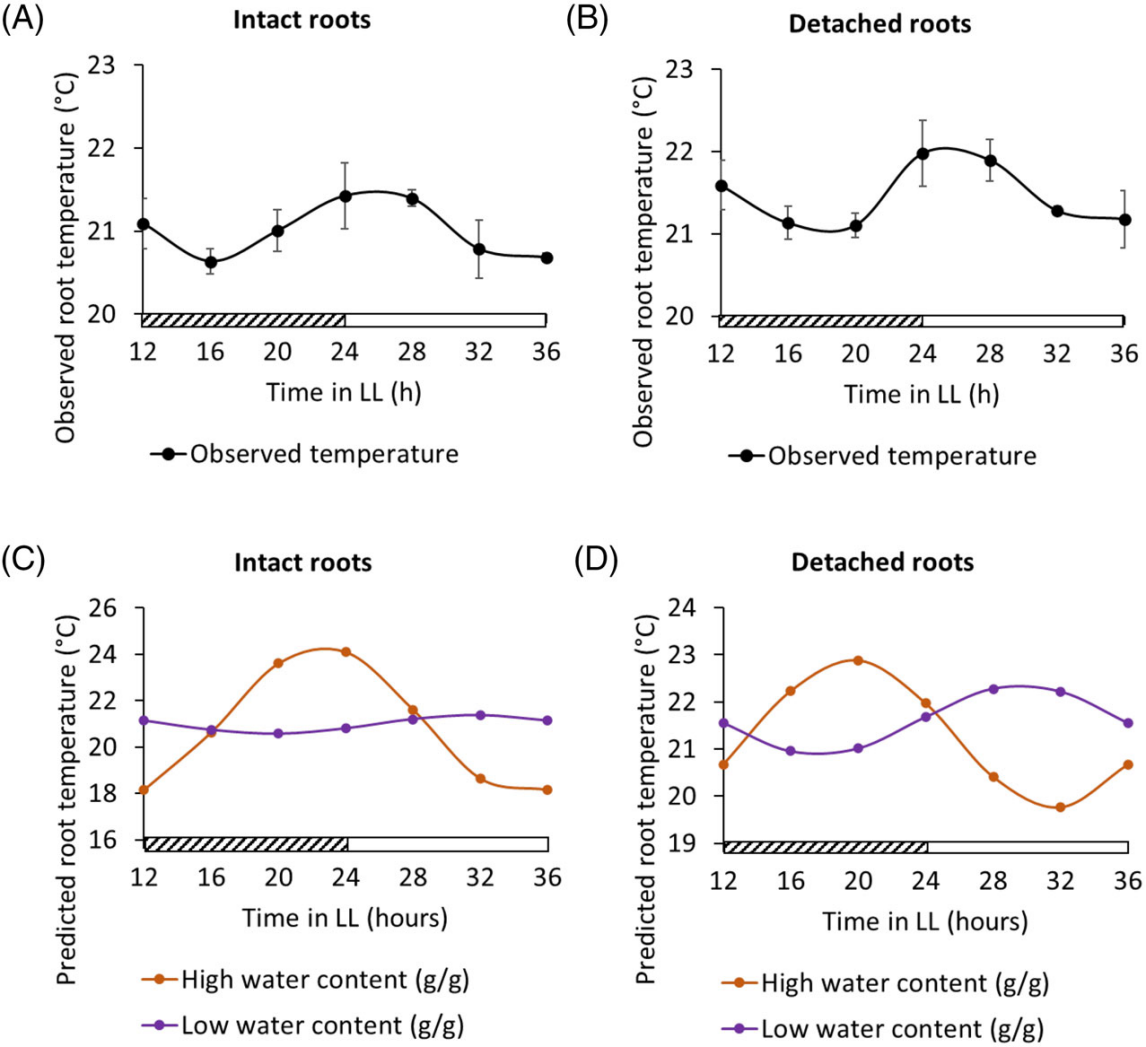
Predicted root temperature across the circadian cycle at low and high soil water content. Tomato plants were grown for 4 weeks under LD conditions, transferred to LL for 24 h either intact or with the roots detached from aerial parts, and root samples were collected every 4 h. Briefly, roots were rinsed, weighed fresh, oven‐dried at 60°C for 48 h, and reweighed. Fresh‐weight and dry‐weight data were obtained from two independent experiments comprising 2–4 plants at each time point per independent repeat, measured across seven time points. Water content data were obtained from two independent repeats with (*n* = 14) for intact and detached roots. Observed root temperature measurements are averages of two independent repeats (*n* = 2) for intact and detached roots. (A, B) Observed root temperature over 24 h in intact (A) and detached (B) roots. (C, D) Thermal oscillations represent model‐predicted values derived from a regression analysis based on the highest and lowest observed water content in intact (C) and detached (D) roots over 24 h (see “Materials and Methods” section for full model and formula). The hatched and white bars represent subjective dark and light, respectively.

To test whether root water content influences circadian oscillations, we measured water content in intact and detached tomato roots across a 24‐h cycle in LL. Plants were entrained under LD conditions, transferred to LL and roots harvested every 4 h for fresh and dry weight measurements. For the detached root measurements, the aerial parts of the plants were excised 24 h before starting to measure. Water content was calculated as [(fresh weight − dry weight)/dry weight], and to examine the relationships between root water content and temperature, we used a statistical model (multiple linear regression with 24‐h sine and cosine terms). The respective models of intact and detached roots were significant (*P* < 0.01, *F* test) (Tables S1 and S2). In both models, root temperature exhibited a significant sine‐based circadian component (*P* < 0.05), whereas the cosine main effect was not significant. The interactions between water content and time [water × sin*time and water × cos*time] were significant (*P <* 0.05), indicating that water content influences how root temperature changes over the day. Models based on the different water content results showed that using the higher water content data produced predictions with pronounced root temperature oscillations patterns, while lower water content showed minimal temporal variation (Figure 7C,D). Observed temperatures in intact roots (Figure 7A) closely followed the high water content prediction (Figure 7C), whereas detached roots (Figure 7B) exhibited peak temperatures that fell between the high and low water content predictions (Figure 7D). The predicted thermal circadian amplitudes were ~eightfold in intact and ~twofold in detached roots higher under the highest water content (Table 4). Taken together our results suggest circadian rhythms of root temperature may be associated with fluctuations in root water content.

**Table 4:** Summary table for results

	Water content (g g^−1^)	Amplitude (°C)	Thermal pattern
High water content (intact roots)	59.4	3.2	Pronounced oscillations
Low water content (intact roots)	2.6	0.4	Minimal temporal variation
High water content (detached roots)	27.4	1.6	Pronounced oscillations
Low water content (detached roots)	9.2	0.7	Minimal temporal variation

## DISCUSSION

In this article, we demonstrate that thermal imaging is a high resolution, non‐invasive method for investigating circadian rhythms in plant roots. The correlations between our thermal imaging results and previously reported features of root circadian rhythms confirm the accuracy of our system. For example, Arabidopsis roots exhibited longer circadian thermal periods than leaves (Bordage et al., 2016; James et al., 2008) and the *ztl* mutant displayed the expected long‐period root phenotype (Li et al., 2020) (Figure 3). Exogenous sucrose is widely used to support circadian rhythms in constant darkness in Arabidopsis, although the concentrations employed vary across studies. For example, ~1% sucrose has been shown to be sufficient to elicit measurable circadian responses in darkness (Li et al., 2022). In contrast, higher concentrations (~3%) are commonly used to sustain more robust oscillations over extended periods (Dalchau et al., 2011). In our experiments, we used a lower concentration (0.5%), which likely provides more limited metabolic support to the oscillator. Consistent with this, we observed progressive damping of thermal rhythmic amplitude after approximately 48 h, reflecting a reduced capacity to sustain oscillations under low‐carbon conditions rather than a complete loss of clock function. Moreover, our results that detached roots maintained thermal circadian properties (Figure 5; Table 3) are consistent with reports that long‐distance signals from the shoot are not imperative for coordination of the root clock (Bordage et al., 2016; Greenwood et al., 2019; Mortada et al., 2024) and show that roots can maintain thermal circadian oscillations independent of the aerial parts of the plant. Finally, the phase differences that are maintained in LL between plants grown in antiphase confirm the accuracy of our thermal imaging system (Figure 2I,J).

One of the strengths of our thermal imaging technique is that it can be used for studying circadian rhythms in roots from a diverse range of plants and under different conditions. Our results showed that roots of different species, including monocots, dicots, and those with C3 or C4 photosynthetic pathways show robust thermal rhythms (Figure 4). In all the species we tested, roots exhibited longer circadian periods than leaves, a trend that appears to hold for most plants though not all, for example, *M. truncatula* have been reported to have shorter period root rhythms (Wang et al., 2021). In addition, we observed that roots showed less variation in circadian period between species (standard deviation = 0.92 h) compared with leaves (standard deviation = 1.88 h), indicating that root rhythms may be more conserved across species. In the future, it will be interesting to investigate whether more generally, non‐photosynthetic tissues exhibit less variation in circadian period than leaves which may suggest an adaptive advantage for photosynthetic tissues to maintain more flexible circadian timing. We also observed significant species‐specific differences in the thermal phase relationships between roots and leaves under light–dark cycles; in tomato and sorghum, root and leaf rhythms were closely phased, whereas in barley and coleus, the two organs showed significant differences in phase (Figure 4B). These differences may be attributed to the divergent evolution of cis‐regulatory elements in circadian regulated genes, leading to distinct circadian phase patterns between species (Castanedo et al., 2025; Marand et al., 2023). Cis‐regulatory elements control the spatial and temporal regulation of gene expression to meet metabolic or developmental demands, such as nutrient uptake or growth, and variations in these elements can produce distinct expression patterns and circadian rhythms in roots and leaves. We have also used our imaging platform to demonstrate the impact of the microbial environment on root circadian traits and suggest there may be synergistic strain‐specific interactions between root‐associated microbes and the plant circadian system. Thus, *Arthrobacter* and *Variovorax* are common bacterial strains known to interact with plant roots and influence their growth (Finkel et al., 2020; Qi et al., 2022), and in our experiments CL14 appeared to counteract the disruptive effect of CL28 on root circadian regulation (Figure 6). However, at this stage, we cannot exclude the possibility that microbial impacts on roots are indirectly affecting thermal rhythms.

Although there are a number of possible causes for circadian changes in root temperature, for example, root growth or heat that may be generated by metabolic cycles (Cervela‐Cardona et al., 2025), our results (Figure 7) suggest that water content modulates the observed 24‐h thermal cycle. While circadian shifts in root water status can be driven by rhythmic changes in transpiration (Nuixe et al., 2021), given our observation that detached roots also exhibit circadian thermal rhythms, water movement from the roots into the drier growth medium may also contribute significantly (Caldwell et al., 1998). Comparable effects of root water content on thermal rhythms in intact and detached roots suggest that the water content changes we observed are likely generated locally within the roots. Moreover, sufficient root hydration may support stronger oscillatory dynamics potentially by facilitating physiological processes like hydrotropism and hydro‐patterning (Bao et al., 2014; Monshausen & Gilroy, 2009). Conversely, the attenuated profiles under water limitation likely reflect constraints such as osmotic stress, loss of cellular turgor, and metabolic impairment (Gargallo‐Garriga et al., 2018; Haghpanah et al., 2024). Collectively, these findings suggest that root water content may influence root temperature rhythms. However, further studies involving direct experimental manipulation of root hydration will be essential to confirm whether root water content is the primary driver of these thermal rhythms.

Finally, we acknowledge that there are technical limitations to the technique as reported here. The high sensitivity of the thermal imaging system requires measurements to be conducted in a closed chamber, which limits the ability to apply treatments or adjust conditions during imaging. Moreover, removing plants from their growth medium risks mechanical damage and exposes roots to light, both of which may influence circadian rhythms. These effects can be mitigated by growing plants on agar‐based media or in hydroponic systems and allowing a short acclimation period prior to imaging, as well as by performing imaging under IR filters that exclude wavelengths below 740 nm (e.g., Optolite IR, Instrument Plastics Ltd, Maidenhead, UK) (Wells et al., 2012). We are confident that although our technique has some practical constraints, many of them can be effectively managed through controlled growth conditions and optimized imaging approaches.

## MATERIALS AND METHODS

### Plant materials and growth conditions

We used *A. thaliana* (Arabidopsis), *S. lycopersicum* (tomato), *S. bicolor* (sorghum), and *H. vulgare* (barley) grown from seed and *C. blumei* (coleus) from cuttings. Tomato and sorghum seeds were purchased from a local nursery, and the wild barley, B1K46, was from (Hübner et al., 2009).

For the Arabidopsis experiments, seeds were sterilized in microcentrifuge Eppendorf tubes with 50% (v/v) of sterile double‐distilled water: 6% Sodium Hypochlorite (Bio Labs) and 10% sodium dodecyl sulfate (SDS) for 8 min. The seeds were then rinsed five times with sterile double‐distilled water, and 7 seeds were sown on the upper portion of 12 × 12 × 1.7 cm square Petri plates (Greiner) containing sterile ½ Murashige and Skoog (MS, Duchefa Biochemie) media (2.2 g MS in 1 L of double‐distilled water) and 0.8% agar (Duchefa Biochemie). The plates were sealed with surgical tape (Micropore, 3M, Deutschland GmbH), and the seeds were stratified at 4°C for 3–4 days. The plates were transferred into growth chambers (Conviron) for the seeds to germinate. Unless otherwise noted, plants were grown in long‐day (14 h:10 h, light:dark; LD) conditions with 150 μmol m^−2^ sec^−1^ white light (Philips fluorescent lights TLD 18W/840). Plates were placed vertically in the growth chambers for the roots to grow downwards across the surface of the media. Arabidopsis ecotype Columbia‐0 (Col‐0) was used as the wild‐type (WT) control and mutants *ztl* (SALK_069091), *elf4* (CS_9386), *cca1‐1 lhy* (SALK_031092) (Yakir et al., 2009), and *CCA1‐ox* (Wang & Tobin, 1998). Unless otherwise stated, Arabidopsis plants were imaged at 3 weeks. To ensure stable and accurate thermal measurements, the chamber remained closed throughout the imaging experiment, and growth conditions were optimized to support the plants over the imaging period. For Arabidopsis plants grown in antiphase, two growth chambers were programmed with light cycles that overlapped by only 4 h. Thus, in Chamber I, lights were switched on at 7:00 (ZT0) and off at 21:00, and in chamber II, lights were switched on at 19:00 (ZT0) and off at 9:00 the following day. This design enabled us to get two entrainment regimes, while maintaining identical environmental conditions. The plants were then transferred to LL and the roots imaged side by side.

Tomato, barley, and sorghum plants were grown in a mixture of commercial soil, vermiculite and perlite (2:1:1) in commercially purchased square pots measuring 8 × 8 cm (Hummert International, USA) under non‐sterile conditions. Barley seeds were stratified at 4°C for 7 days before being transferred to a growth chamber at 22°C. The plants were watered once or twice every week, according to need. Coleus experiments were carried out on plant cuttings made by cutting stems just below a node, removing all but the top 3 or 4 leaves and placing them into 50 ml Centrifuge tubes (Mini‐plast Ein‐Shemer) filled with water, with the cut ends of the plants submerged in water. At 4 weeks, the roots were long enough for imaging. All the plants were grown in 14 h light:10 h dark (LD; 150 μmol m^−2^ sec^−1^ white Philips fluorescent lights TLD 18W/840). All experiments were done at a constant 22°C. Unless otherwise stated, tomato, sorghum, barley and coleus were imaged after 4 weeks.

For experiments involving detached roots, scissors were used to cut through the root–shoot junction of 3‐week‐old Arabidopsis, or 4‐week‐old tomato, sorghum, coleus, and barley plants previously grown in 14 h:10 h LD conditions. The upper section of the roots was wrapped in plastic wrap to preserve moisture and secured with adhesive tape. The middle portions of the roots were left exposed for imaging, and the root tips were submerged in water.

### Microbial treatments for Arabidopsis plants

Bacteria strains *Variovorax* CL14 and *Arthrobacter* CL28 (Finkel et al., 2020) were taken from stock cultures stored at −80°C into 3 ml of Luria broth (LB) medium (10 g L^−1^ Tryptone, 5 g L^−1^ yeast extract and 10 g L^−1^ NaCl, pH of 7.0 with NaOH). The liquid bacteria cultures were grown in an incubator (New Brunswick classic C24 Incubator shaker) at 28°C shaking at 250 revolutions per minute (rpm). The cultures were then washed twice with 10 mM MgCl_2_, centrifuging at 6500 rpm for 4 min to remove spent media and cell debris. The optical density of cultures was measured at 600 nm (OD_600_) and each culture sample normalized to OD_600_ = 0.02. To prepare a bacterial combination of CL14 and CL28, each strain was independently cultured and normalized to OD_600_ = 0.02. Each 12 × 12 × 1.7 cm square Petri plate (Greiner) containing solid ½ MS was inoculated with either 200 μl of MgCl_2_, 200 μl of an individual bacterial strain or a 200 μl bacteria mixture prepared by combining 100 μl of each bacterial suspension, evenly spread across the plate. Two‐week old Arabidopsis plants grown on ½ MS in LD were transferred to the bacteria/ MgCl_2_ plates and arranged in a single row at the top edge of the plate, about six plants on each plate. The plants were incubated for 6 days in LD at 22°C before imaging in LL. All the work involving bacteria and Arabidopsis on plates was performed in sterile conditions to avoid cross‐contamination.

### Preparation of plants for thermal imaging

For imaging the roots of plants grown in pots, we gently pulled them out of the soil then washed the roots in a slow stream of tap water until they were completely free of soil. To remove plants from the MS/agar media, we added 1 ml of water to the agar for 5 min to make it easier to detach the plant roots from the medium. The roots were then straightened and untangled to facilitate a clearer view of the root system and were then cleaned again with tap water. The plants were placed on a black surface with the root tips submerged in water or 0.5% sucrose solution (Figure 1). The black surfaces were cut from black plant pots or black seedling bags and then secured with adhesive paper tape onto an empty small plastic box. The roots were gently bound together with a small piece of adhesive tape. Any gaps between the water and the surrounding environment were sealed with plastic wrap to minimize temperature fluctuations and prevent rapid evaporation from the uncovered container (Figure 1A–C). To ensure a stable temperature throughout the experiment, the growth chamber was kept closed. It was therefore important to ensure that the plants had sufficient water before the imaging was started.

Leaf thermal imaging was performed as described by Dakhiya and Green (2019) except that Arabidopsis leaves were also secured on the black surfaces using small pieces of adhesive tape to minimize movement during multiday imaging.

### Thermal imaging of root and leaf circadian rhythms

Temperature measurements were taken either in 14:10 h Light–Dark (LD), continuous light (LL) at an intensity of 150 μmol m^−2^ sec^−1^ light, with 50% red light (615 nm) and 50% blue light (450 nm), or continuous dark (DD) conditions at a constant temperature of 22°C largely as described in by Dakhiya and Green (2019). The plants for circadian thermal measurements were placed in a customized Fytoscope FS‐FI‐2200 growth chamber from Photon System Instruments (PSI, Drasov, Czech Republic) and acclimatized for 24 h prior to imaging. The chamber maintains temperature within a 0.3°C range of the set point but does not control humidity or carbon dioxide concentrations.

Thermal imaging was conducted using a FLIR‐05A305sc thermal imaging camera from FLIR systems (Täby, Sweden) that can detect temperature variations as small as 50 mK. The camera features an 18 mm lens, a resolution of 320 × 240 pixels, a 9 Hz frame rate, and a noise equivalent temperature difference (NETD) of 50 mK. The camera was calibrated following the manufacturer's guidelines and positioned above the plants within the measurement chamber. The distance between the camera and the plants was adjusted based on the plant type and the size of the area being measured. Air temperature was recorded every 5 min using a D Logmate TD detector from MRC (Holon, Israel) placed inside the chamber. To ensure consistency in temperature measurements, the thermal radiation setting (emissivity) for both roots and leaves was set to a fixed value of 0.95. Thermal images were captured at 5‐min intervals over a period of up to 5 days to monitor temperature dynamics with high temporal resolution. To control for temperature fluctuations in the chamber we included a piece of aluminum foil placed 10 cm from the plants. The low heat capacity of the aluminum makes it a useful reference for technical temperature changes to allow us to normalize the leaf and root temperatures (root or leaf temperature: aluminum foil temperature). Figure S1 shows temperature profiles captured by aluminum foil standards under LL conditions in the growth chamber.

The normalized root and leaf temperatures were analyzed using the BioDare2 Analysis Software Program (https://biodare2.ed.ac.uk/) (Zielinski et al., 2014). Rhythms with a period between 18 and 34 h were taken to be within the circadian range. We opted to use the Spectrum Resampling (SR) suite of the BioDare2 Analysis Software Program, as previously described by Costa et al. (2013). The 5‐min sampling interval we chose for root and leaf thermal imaging allows us to capture fine‐scale temperature fluctuations, ensuring accurate detection of low‐amplitude circadian rhythms and reducing the risk of missing transient changes which improves the reliability of period estimation. However, this finer sampling also increases likelihood of noise and irregular waveforms, which traditional Fourier‐based methods might fail to accurately capture. For example, in the sorghum leaves, low‐amplitude rhythms were visually detectable and captured by SR but were not identified by non‐uniform fast Fourier transform (NFFT). From the SR analysis we obtained period, amplitude, rhythmic fraction (RF), phase and relative amplitude error (RAE) data. RF indicates the number of random measurement points of plant leaves or roots that showed rhythmicity. RAE represents the ratio of amplitude error to amplitude to serve as a measure of circadian rhythm accuracy. An RAE of 1 represents the most irregular waveform that can still be classified as rhythmic, while an RAE of 0 corresponds to a perfect sine wave with no amplitude error. Typically, values below 0.7 indicate robustly accurate circadian rhythms in Arabidopsis (Plautz et al., 1997).

### Dry and wet root weight measurements

Tomato plants were grown in 14:10 h LD conditions in soil for 4 weeks before being transferred to LL. For experiments involving detached roots, scissors were used to cut through the root–shoot junction of 4‐week‐old tomato plants previously grown in 14 h:10 h LD conditions. The upper section of the roots was wrapped in plastic wrap to preserve moisture and secured with adhesive tape. After 1 day in LL, every four 4‐h for 24‐h, roots were extracted from the soil, thoroughly rinsed with clean water to remove adhering particles, and immediately weighed. The roots were then dried in an oven (BINDER GmBH, Germany) at 60°C for 48 h and reweighed.

### Statistical modeling of root thermal rhythms and water content

To assess the interaction between root thermal oscillations and water content, we employed a multiple linear regression model with transformed periodic variables. Time (*t*) was linearized into sine and cosine components: sin*time = Sin(2π*t*/24) and cos*time = cos(2π*t*/24). To specifically test if water content modulates the thermal rhythm, we included the interaction terms: Water × sin*time and Water × cos*time. The final model was defined as:

$$Y=\beta_{0}+\beta_{1}\;\left(\mathrm{Sin}\right)+\beta_{2}\;\left(\mathrm{Cos}\right)+\beta_{3}\;\left(\text{Water}\right)+\beta_{4}\;\left(\text{Water}\times \mathrm{Sin}\right)+\beta_{5}\;\left(\text{Water}\times \mathrm{Cos}\right)+\varepsilon$$

where *β*_0_ is the intercept representing the baseline level of the temperature. *β*_1_ and *β*_2_ are the coefficients of the sine and cosine terms, respectively, and together describe the amplitude and phase of the 24‐h thermal rhythmic variation. *β*_3_ represents the main effect of water content on the mean level temperature. *β*_4_ and *β*_5_ represent the interaction effects between water and the sine and cosine components, respectively, while *ε* is the error term which represents random variation in root temperature. This approach allowed us to simultaneously test if water influences the average level (MESOR) of the thermal response and if it significantly alters the amplitude and/or phase of the 24‐h circadian oscillation.

To generate the predicted thermal profiles (Figure 7), we varied time (*t*) from 12 to 36 h while keeping the respective maximum and minimum water content for intact and detached roots. We then calculated the predicted temperature at every time point throughout the circadian cycle with the formula

$$T_{\text{predicted}}\;\mathrm{at}\;\text{maximum water content}=\beta_{0}+\beta_{1}\;\left(\sin^{*}\text{time}\right)+\beta_{2}\;\left(\cos^{*}\text{time}\right)+\beta_{3}\left(\max\right)+\beta_{4}\left(\max\times \sin^{*}\text{time}\right)+\beta_{5}\;\left(\max\times \cos^{*}\text{time}\right)$$

$$T_{\text{predicted}}\;\mathrm{at}\;\text{minimum water content}=\beta_{0}+\beta_{1}\;\left(\sin^{*}\text{time}\right)+\beta_{2}\;\left(\cos^{*}\text{time}\right)+\beta_{3}\left(\min\right)+\beta_{4}\left(\min\times \sin^{*}\text{time}\right)+\beta_{5}\;\left(\min\times \cos^{*}\text{time}\right)$$

Max or min refers to the maximum or minimum observed water content in intact or detached roots. Sin*time and cos*time components represent the time of day (12–36 h) in radians, calculated by scaling time to a 24‐h cycle. Circadian amplitude [√ (*β*_cos^2^ + *β*_sin^2^)] for both low and high water content was calculated from the combined sine and cosine coefficients.

## CONFLICT OF INTEREST

The authors declare no conflict of interest.

## Supporting information


**Figure S1.** To monitor fluctuations in the growth chamber temperature, the temperature of four pieces of aluminum foil was measured. The foil was positioned within the camera frame, about 20 cm from the plants. The black line represents a 10‐point moving average from three separate experiments. White and hatched bars represent subjective light and dark periods, respectively.
**Tables S1 and S2.** Multiple regression analysis of the effect of root water content on root thermal rhythms of intact and detached roots.

## Data Availability

Data available on request from the authors.
