# Supplementary material for: Thermal imaging as a tool for studying circadian rhythms in roots
Source: Plant J. 2026 Jul 8;127(1):e70992. doi: 10.1111/tpj.70992 (PMC13345701; doi:10.1111/tpj.70992)
Supplement: Supplementary file 1 — Figure S1. To monitor fluctuations in the growth chamber temperature, the temperature of four pieces of aluminum foil was measured. The foil was positioned within the camera frame, about 20 cm from the plants. The black line represents a 10‐point moving average from three separate experiments. White and hatched bars represent subjective light and dark periods, respectively. Tables S1 and S2. Multiple regression analysis of the effect of root water content on root thermal rhythms of intact and detached roots. [file TPJ-127-0-s001.docx]

**Supplementary data**


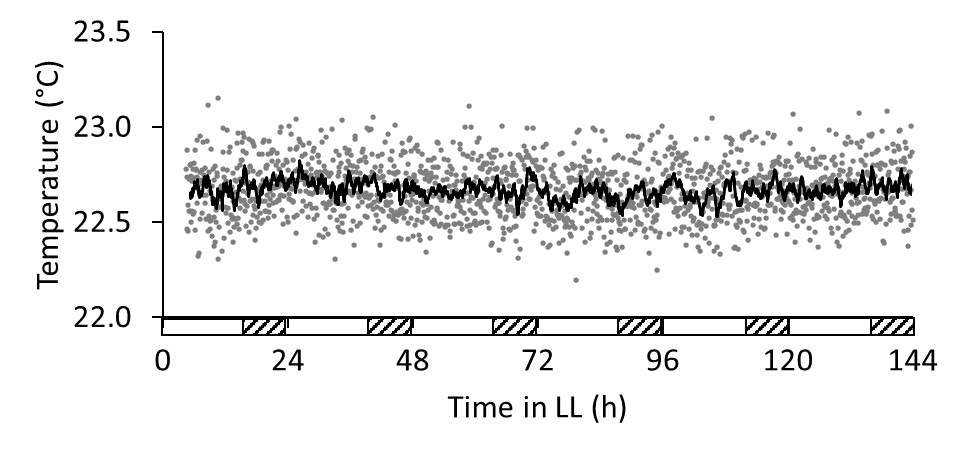


**Supplementary Figure S1**: To monitor fluctuations in the growth chamber temperature, the temperature of 4 pieces of aluminium foil was measured. The foil was positioned within the camera frame, about 20 cm from the plants. The black line represents a 10-point moving average from 3 separate experiments. White and hatched bars represent subjective light and dark periods, respectively.

**Supplementary Tables S1 and S2. Multiple regression analysis of the effect of root water content on root thermal rhythms of intact and detached roots.**

The model includes 24- hour periodic rhythmic components represented by sine and cosine transformations of time: sin*time and cos*time, along with their interactions with water content. The interaction terms [water (sin*time)] and [water (cos*time)] represent the modulation of the rhythmic profile by water content. The table reports regression coefficients, standard errors, t-values, and p-values for each term.

**Supplementary Table S1**

| SUMMARY OUTPUT FOR INTACT ROOTS | | | | |  |
| --- | --- | --- | --- | --- | --- |
|  |  |  |  |  |  |
| *Regression Statistics* | |  |  |  |  |
| Multiple R | 0.92292 |  |  |  |  |
| R Square | 0.85178 |  |  |  |  |
| Adjusted R Square | 0.75914 |  |  |  |  |
| Standard Error | 0.15252 |  |  |  |  |
| Observations | 14 |  |  |  |  |
|  |  |  |  |  |  |
| ANOVA |  |  |  |  |  |
|  | *df* | *SS* | *MS* | *F* | *Significance F* |
| Regression | 5 | 1.06946 | 0.21389 | 9.19453 | 0.00361 |
| Residual | 8 | 0.1861 | 0.02326 |  |  |
| Total | 13 | 1.25556 |  |  |  |
|  |  |  |  |  |  |
|  | *Coefficients* | *Standard Error* | *t Stat* | *P-value* |  |
| Intercept | 20.9656 | 0.0899 | 233.203 | 1.3E-16 |  |
| Sin*time | 0.43081 | 0.16413 | 2.6248 | 0.03042 |  |
| Cos*time | -0.3094 | 0.20803 | -1.4872 | 0.17528 |  |
| Water content | 0.00266 | 0.00504 | 0.52862 | 0.61142 |  |
| Water (sin*time) | -0.0268 | 0.0113 | -2.3679 | 0.04539 |  |
| Water (cos*time) | 0.0554 | 0.01832 | 3.02457 | 0.01644 |  |

**Supplementary Table S2**

| SUMMARY OUTPUT FOR DETACHED ROOTS | | | | |  |
| --- | --- | --- | --- | --- | --- |
|  |  |  |  |  |  |
| *Regression Statistics* | |  |  |  |  |
| Multiple R | 0.928092 |  |  |  |  |
| R Square | 0.861356 |  |  |  |  |
| Adjusted R Square | 0.774703 |  |  |  |  |
| Standard Error | 0.167969 |  |  |  |  |
| Observations | 14 |  |  |  |  |
|  |  |  |  |  |  |
| ANOVA |  |  |  |  |  |
|  | *df* | *SS* | *MS* | *F* | *Significance F* |
| Regression | 5 | 1.402266 | 0.280453 | 9.940313 | 0.002796 |
| Residual | 8 | 0.22571 | 0.028214 |  |  |
| Total | 13 | 1.627976 |  |  |  |
|  |  |  |  |  |  |
|  | *Coefficients* | *Standard Error* | *t Stat* | *P-value* |  |
| Intercept | 21.76703 | 0.172123 | 126.4618 | 1.71E-14 |  |
| Sin*time | 1.81009 | 0.53839 | 3.362044 | 0.009902 |  |
| Cos*time | -0.23634 | 0.192198 | -1.22969 | 0.253747 |  |
| Water content | -0.01634 | 0.012305 | -1.32798 | 0.220822 |  |
| Water (sin*time) | -0.11785 | 0.042361 | -2.78197 | 0.023852 |  |
| Water (cos*time) | 0.032336 | 0.012949 | 2.497146 | 0.037107 |  |
